# Selection for feed efficiency in Atlantic salmon using individual indicator traits based on stable isotope profiling

**DOI:** 10.1186/s12711-019-0455-9

**Published:** 2019-04-15

**Authors:** Hanne Dvergedal, Jørgen Ødegård, Margareth Øverland, Liv Torunn Mydland, Gunnar Klemetsdal

**Affiliations:** 1 Department of Animal and Aquacultural Sciences, Faculty of Biosciences, Norwegian University of Life Sciences, Post Box 5003, 1433 Ås, Norway; 2 AquaGen AS, Post Box 1240, 7462 Trondheim, Norway

## Abstract

**Background:**

We used stable isotope profiling (^15^N and ^13^C) to obtain indicator phenotypes for feed efficiency in aquaculture. Our objectives were to (1) examine whether atom percent of stable isotopes of nitrogen and carbon can explain more of the variation in feed conversion ratio than growth alone, and (2) estimate the heritabilities of and genetic correlations between feed efficiency, growth and indicator traits as functions of nitrogen and carbon metabolism in various tissues. A 12-day experiment was conducted with 2281 Atlantic salmon parr, with an average initial weight of 21.8 g, from 23 full-sib families that were allocated to 46 family tanks and fed an experimental diet enriched with ^15^N and ^13^C.

**Results:**

Using leave-one-out cross-validation, as much as 79% of the between-tank variation in feed conversion ratio was explained by growth, indicator traits, and sampling day, compared to 62% that was explained by growth and sampling day alone. The ratio of tissue metabolism, estimated by a change in isotope fractions relative to body growth, was used as an individual indicator for feed efficiency. For these indicator ratio traits, the estimated genetic correlation to feed conversion ratio approached unity but their heritabilities were low (0.06 to 0.11). These results indicate that feed-efficient fish are characterized by allocating a high fraction of their metabolism to growth. Among the isotope indicator traits, carbon metabolism in the liver had the closest estimated genetic correlation with feed conversion ratio on a tank level (− 0.9) but a low estimated genetic correlation with individually recorded feed efficiency indicator ratio traits. The underlying determinants of these correlations are largely unknown.

**Conclusions:**

Our findings show that the use of indicator ratio traits to assess individual feed efficiency in Atlantic salmon has great prospects in selection programs. Given that large quantities of feeds with contrasting isotope profiles of carbon and/or nitrogen can be produced cost-effectively, the use of stable isotopes to monitor nitrogen and carbon metabolism in various tissues has potential for large-scale recording of individual feed efficiency traits, without requiring individual feed intake to be recorded.

## Background

The steadily growing human population increases the demand for protein resources from both the livestock and aquaculture industries. In 2050, the number of mouths to feed is expected to reach ~ 9 billion [1]. In the near future, livestock and aquaculture production will be in competition with direct human consumption for many of the same protein resources and, therefore, efficiency must be increased. Selective breeding is, and has for several decades, been an important tool to improve feed efficiency in both livestock and farmed fish [2–6].

Feed efficiency can be defined as feed conversion ratio (FCR), which is the amount of feed consumed per unit growth, or alternatively, by its inverse, the feed efficiency ratio (FER), i.e., growth per unit of feed consumed [7]. Selective breeding for improved feed efficiency assumes that both individual growth and individual feed intake can be routinely recorded on a large number of individuals. In aquaculture, recording of individual growth rate is easily attainable, and it has been the major trait in breeding schemes of Atlantic salmon since the 1970s [8]. Various methods for recording individual feed intake have been proposed such as X-radiography, where generally radio-opaque ballotini glass beads are mixed into the feed, fish are X-rayed, and the number of pellets eaten is counted [9–12]. Video recording is another method for feed intake recording [13, 14], with manual feeding of pellets one by one and retrospective identification of individual fish from video analysis. However, since sib-testing of Atlantic salmon is carried out in large sea-cage units and since fish are communally fed with feed dispersed into the water, large-scale recording of individual feed intake with these methods is difficult to implement in selective breeding programs of Atlantic salmon. Hence, the first option in selective breeding for improved FCR has been to rely on selection for traits such as growth rate [15, 16], which has been shown to improve feed retention ratio and FCR [3, 16–19] because of the generally accepted high genetic correlation between FCR and growth rate, ranging from 0.63 to 0.99 in rainbow trout (*Oncorhynchus mykiss*) [20]. The effect of increased growth rate on feed efficiency is through reducing maintenance requirements per unit of growth produced, mainly by reducing time to slaughter. Kause et al. [12] proposed to add information from indicator traits such as the percentage of muscle lipid to enhance the genetic progress in feed efficiency, which could be an alternative to recording feed intake.

In our study, we examined the potential use of stable isotopes to assess feed efficiency traits in Atlantic salmon, with the objective to establish indicator phenotypes that explain more of the genetic variation in feed efficiency than growth alone. McCarthy et al. [9] identified individual variation in protein metabolism, with feed efficient fish having a lower protein degradation for the same level of feed eaten than inefficient fish. The potential use of feeding stable isotope such as ^15^N to fish to assess individual protein metabolism was investigated in a previous study [21]. In this study, fish were fed a standard diet (low in ^15^N) followed by a ^15^N-enriched diet with various inclusion levels, which resulted in isotope profile changes of body nitrogen (protein metabolism), which is closely related to body growth. Using protein-bound ^15^N enrichment, significant correlations between relative weight gain and protein metabolism were found in muscle (*r* = 0.31–0.98) and in liver (*r* = 0.59–0.94) [21]. This study also found that not all individual variation in protein metabolism was explained by growth. Isotope profiles can be recorded individually, in contrast to the challenge of recording feed intake and feed efficiency at the individual level. If feed efficiency can be accurately predicted by atom percentages (atom%) of nitrogen and carbon stable isotopes, individual isotope profiles could be used for more direct selection for improved feed efficiency. However, first it is necessary to validate the method in an experiment in which both isotope profiles and feed efficiency are recorded and estimate associated genetic parameters, i.e., in family material. This requires a large-scale experiment, in which families are kept in separate (replicate) tanks, and feed consumption and growth are monitored at the tank level. Dvergedal et al. [21] reported a curvilinear increase in the level of isotopes in tissue over time, with the atom% reaching an asymptote when fish were fed until saturation, i.e., all fish will eventually approach equilibrium isotopic levels, reflecting that of the feed. This implies that length of the experiment is crucial for recording individual variation in metabolism, since individual variation in nitrogen and carbon metabolism can be detected only prior to the point when the fish are expected to be in equilibrium with the isotopic level in the feed.

In this paper, we report the results of a large-scale experiment, in which families were kept separate in replicate tanks, growth and isotope profiles were recorded at the individual level, and feed consumption and FCR were recorded at the tank level. Feed was labelled with both ^15^N and ^13^C stable isotopes. One objective was to examine whether the atom% of stable isotopes of nitrogen and carbon can explain more of the variation in FCR than growth alone, i.e. to explore the potential of using indicator traits in selective breeding for improved feed efficiency in Atlantic salmon. Another objective was to estimate the heritabilities of and genetic correlations between feed efficiency, growth and indicator traits, as functions of nitrogen and carbon metabolism in various tissues.

## Methods

### Fish and housing

The experiment included 23 full-sib families (offspring of 23 dams and 22 sires) of Atlantic salmon (*Salmo salar*) from AquaGen’s breeding population. To ensure clearly contrasted family groups with respect to growth potential and, potentially, feed efficiency, the parents of the families were selected for high/low estimated breeding values for growth in seawater, although the experiment was conducted in freshwater.

From the eyed egg stage until the start of the experiment, all families were communally reared in a single tank. Before pit-tagging, 15 fish were individually weighed to establish whether they were ready for tagging. The fish were pit-tagged with a 2 × 12 mm unique glass tag (RFID Solutions, Hafrsfjord, Norway) and a fin-clip was collected for genotyping. All fish were genotyped using AquaGen’s custom Axiom^®^SNP genotyping array from Thermo Fisher Scientific (San Diego, CA, USA), which includes 56,177 single-nucleotide polymorphisms (SNPs). Prior to the experiment, the parentage of each individual fish was established using genomic relationship likelihood for parentage assignment [22].

Based on parentage assignment, 100 family members were identified for each of the 23 families used in the experiment. These fish were randomly allocated to family tanks with 50 fish per tank and two tanks per family, except for nine tanks in which the number of fish varied between 42 and 54, due to some mortality prior to the start of the experiment and to a larger number in one tank because of a counting mistake. A single fish was allocated to an incorrect family tank but it was later identified. In total, 2281 fish were included in the experiment. The tanks, each with a 270-L capacity, were supplied with recirculated fresh water, at a flow rate of 7 to 8 L.min^−1^, and the fish were kept under 24 h light regime, with an average temperature of 14.5 °C. Dissolved oxygen was measured daily and maintained above 8 mg.L^−1^ in the outlet water (Handy Delta, OxyGuard^®^ AS, Farum, Denmark).

### Dietary treatment and feeding

A labelled diet with the stable isotopes ^15^N and ^13^C, with inclusion levels of 2% and 1% respectively, was fed during the experimental period of 12 days. Due to the large variation in growth rate and thus in the rate of inclusion of new nutrients among families, a pre-defined period of 12 days was set to feed the labelled feed, such that an equilibrium was not reached in any of the families. Termination of the experiment and tissue sampling were done over a 5-day period with different tanks being sampled each day, i.e., the dietary switch was done according to the pre-defined termination day of the tank. The formulation and analysed chemical composition of the diet are in Table 1. The diet was produced at the feed laboratory of the Norwegian University of Life Sciences, Aas, Norway, as explained by Dvergedal et al. [21]. The fish were fed twice daily (07:00 and 15:00) for a period of 1 h, by automatic belt feeders. The feeding level equalled 10% in excess, based on the level of uneaten feed. Registrations of uneaten feed and calculations of feed intake were performed according to Helland et al. [23]. The daily feed intake per tank was calculated by first collecting the waste feed on a wedge wire screen [24] and correcting the total waste feed for leasing losses. As explained by Shomorin et al. [24], the wedge wire is placed at an inclined position in the outlet water column of the tank. The design of the screen ensures efficient drainage so that uneaten feed that is trapped on the screen is exposed minimally to water. Then, the difference between total fed feed and total uneaten feed was calculated as g dry matter intake, after drying the uneaten feed at 105 °C overnight.

**Table 1:** Formulation and analysed content^a^ of the experimental diet

	Content
Formulation (g kg^−1^)	
Fish meal^b^	455.8
Gelatinized potato starch^c^	105.9
Wheat gluten^d^	150.0
Spirulina ^15^N-labelled^e^	20.0
Spirulina ^13^C-labelled^f^	10.0
Fish oil^g^	170.0
Gelatine^h^	80.0
Premix fish^i^	6.3
Monocalcium phosphate^j^	2.0
Analysed content (kg^−1^)	
Dry matter (g)	912.5
Crude protein (g)	512.7
Lipid (g)	187.3
Starch (g)	103.7
Ash (g)	75.6
Gross energy (MJ)	22.2
Analysed content (%)	
Atom ^15^N	2.7^k^
Atom ^13^C	2.0^l^
Essential amino acids (g kg^−1^)	
Arginine	30.3
Histidine	8.8
Isoleucine	19.6
Leucine	34.6
Lysine	28.2
Methionine	11.2
Phenylalanine	20.0
Threonine	19.2
Valine	23.0
Tryptophan	4.1
Non-essential amino acids (g kg^−1^)	
Alanine	31.6
Aspartic acid	39.2
Glycine	43.7
Glutamic acid	99.4
Cysteine	4.8
Tyrosine	11.9
Proline	39.9
Serine	24.2
Total amino acids	493.7

^a^Analysis performed in duplicates

^b^Norse LT 16-001, Norsildmel, Egersund Sildoljefabrikk AS, Egersund, Norway

^c^Lygel F 60, Lyckeby Culinar, Fjälkinge, Sweden

^d^Vital Wheat Gluten, Amilina, Panevezys, Lithuania

^e^CIL-NLM-8401 Spirulina Whole cells (U-^15^N, 98% +), Cambridge Isotope Laboratories, Larodan, Solna, Sweden

^f^CIL-CLM-8400 Spirulina Whole cells (U-^13^C, 98% +), Cambridge Isotope Laboratories, Larodan, Solna, Sweden

^g^NorSalmOil, Norsildmel, Bergen, Norway

^h^Rousselot^®^ 250 PS, Rousselot SAS, Courbevoie, France

^i^Farmix, Trouw Nutrition, LA Putten, the Netherlands. Per kg feed; retinol 2500.0 IU, cholecalciferol 32400.0 IU, α-tocopherol SD 0.2 IU, menadione 40.000 mg, thiamine 15.0 mg, riboflavin 25.0 mg, d-Ca-pantothenate 40.002 mg, niacin 150.003 mg, biotin 3000.0 mg, cyanocobalamin 20.0 mg, folic acid 5.0 mg, pyridoxine 15.0 mg, ascorbate polyphosphate 0.098 g, Cu: Cu sulfate 5H_2_O 11.998 mg, Zn: Zn sulfate 89.992 mg, Mn: Mn(II) sulfate 34.993 mg, I: K-iodine 1.999 mg, Se: Na-selenite 0.200 mg, Cd Max. 0.0003 mg, Pd Max. 0.028 mg, Ca 0.915 g, K 1.380 g, Na 0.001 g, Cl 1.252 g

^j^Bolifor^®^MCP-F.KPP Oy, Animal Nutrition, Helsingborg, Sweden

^k^SE = 0.1

^l^SE = 0.02

### Sampling

Sampling was carried out over 5 days, about 10 tanks were sampled each day, i.e. ~ 500 fish daily. Fish were anesthetized with metacaine (MS-222TM; 1 g.L^−1^ water) and killed with a sharp blow to the head prior to dissection. Whole body weight and length were recorded for all fish, and tissue samples from muscle, liver, and adipose were collected in a cryotube, snap-frozen in liquid nitrogen and stored at − 20 °C until stable isotope analysis. Tissue sampling was standardized; muscle was sampled in the front area of the dorsal fin (1 × 1 cm cube), the liver was divided into four small pieces, and adipose tissue was sampled from the fat that was deposited around the gut between the pyloric ceca and the distal intestine.

### Chemical analysis

The feed was dried and ground prior to analysis, and analyses were performed in duplicate for dry matter by drying to a constant weight at 104 °C, for ash by combustion at 550 °C, for crude protein by Kjeldahl nitrogen × 6.25 according to Commission Regulation (EC) No 152/2009, and for starch as described in McCleary et al. [25]. Lipid was determined after extraction with petroleum ether and acetone (70/30) on an accelerated solvent extractor (ASE 200) (Dionex Corp, Sunnyvale, CA, USA), while gross energy was established with a PARR 1281 Adiabatic bomb calorimeter (Parr Instruments, Moline, IL, USA) according to ISO 9831. Amino acids were analysed according to Commission Regulation (EC) No 152/2009, for all amino acids except tryptophan, on a Biochrom 30 amino acid analyser (Biochrom Ltd,. Cambridge, UK). Tryptophan was analysed according to Commission Regulation (EC) No 152/2009 with a Dionex Ultimate 3000 HPLC system (Dionex Softron GmbH, Germering, Germany) and a Shimadzu RF-535 fluorescence detector (Shimadzu Corporation, Kyoto, Japan).

### Stable isotope analysis

Tissue samples were freeze-dried and homogenized, and samples of approximately 1 mg were weighed into small tin capsules (8 x 5 mm, Elemental Microanalysis, Devon, UK). Samples were analysed for N- and C-isotope compositions using a Nu Horizon isotope-ratio mass spectrometer (IRMS) (Nu Instruments, Wrexham, UK) coupled to a Eurovector element analyser (EA) 3028 (Eurovector S.p.A, Redavalle, Italy) at the Institute for Energy Technology (Kjeller, Norway). Analysed contents of ^15^N and ^13^C in the diet are in Table 1.

Isotopic signatures were reported as $\delta$ values, and Atom% was calculated as follows (taking ^15^N as an example) [26]:

$${\text{Atom\% }}^{15} {\text{N}} = \left( {\frac{{\left( {\delta^{15} {\text{N}}_{Sample} + 1000} \right)}}{{\left( {\delta^{15} {\text{N}}_{Sample} + 1000 + \left( {\frac{1000}{{\delta^{15} {\text{N}}_{Standard} }}} \right)} \right)}}} \right) 100,$$

where $\delta^{15} {\hbox{N}}_{Sample}$ ($\delta^{13} {\text{C}}_{Sample}$) and $\delta^{15} {\text{N}}_{Standard}$ $(\delta^{13} {\text{C}}_{Standard}$) are the proportion of ^15^N divided by the proportion of ^1^^4^N in the sample and in the reference standard (air for nitrogen; $\delta^{15} {\text{N}}_{Standard}$ = 0.003676 [27], and Vienna Pee Dee Belemnite for carbon (VPDB); $\delta^{13} {\text{C}}_{Standard}$ = 0.0112372 [28]). The atom% ^15^N and ^13^C in excess (APE) after feeding with enriched feed is proportional to the fraction of newly deposited amino acids in the tissue, resulting from both tissue growth and replacement of previously deposited nitrogen and carbon, denoted as metabolism. Atom% ^15^N (^13^C) in excess is the total atom% ^15^N (^13^C) in the sample adjusted for the initial isotope percentage in the sample (IA%). Initial isotope profile was accounted for in the calculations of individual feed conversion ratio (IFCR) and of individual feed efficiency ratio (IFER) (described in the next paragraph). Prior to the experiment IA% was assessed by using 20 randomly sampled fish from the experimental population. The ^15^N average and standard deviations were 0.370 ± 0.0001 in muscle and 0.370 ± 0.0003 in liver. Corresponding values for ^13^C in muscle, liver, and adipose tissue were 1.087 ± 0.0005, 1.086 ± 0.0007 and 1.082 ± 0.0003, respectively.

Calibration of ^15^N and ^13^C was performed against international certified reference materials and internal standards. The internal standard IFE Trout and USGS-41 were analysed as unknowns, and certified standards such as USGS-41 (certified value), IAEA (International Atomic Energy Agency) N-1, USGS-24, Isolife P10501 and IAEA 311 were used to define the calibration curve. Three calibration standards (USGS-41, USGS-24, and Isolife P10501) were analysed in each sequence, with ~ 60 samples per sequence. In addition, IAEA 303B ($\delta$ ^13^C_VPDB_: 466 ± 3) was analysed on multiple occasions to verify the linearity of $\delta$ ^13^C_VPDB_ measurements above the Isolife P10501 standard. The $\delta$ ^15^N composition of IFE trout was calibrated using a two-point calibration curve using IAEA 311 and IAEA-N-1 standards. The $\delta$ ^13^C composition of IFE trout was calibrated against the USGS-24 standard. The average $\delta$ ^15^N in IFE trout was 11.60‰ with a standard deviation of 0.20 and, correspondingly, for $\delta$ ^13^C the average was − 20.22‰ with a standard deviation of 0.19. The corresponding $\delta$ ^15^N values for samples analysed according to IAEA 311 were 4693 ± < 5.0‰, and for $\delta$ ^13^C values according to USGS-24 the values were  − 16.05 ± < 0.25‰.

### Phenotypes analysed

When entering the tank, the initial weight of each fish $i$ ($IW_{i}$, g) was recorded. After the experiment, i.e. at sampling, final weight ($FW_{i}$, g) was recorded. From these two variables, individual weight gain ($WG_{i}$) and relative weight gain ($RG_{i}$) were calculated as follows:

$$\begin{aligned} WG_{i} & = FW_{i} - IW_{i} , \\ RG_{i} & = \left( {\left( {FW_{i} - IW_{i} } \right)/FW_{i} } \right) \times 100. \\ \end{aligned}$$

A total of 32 fish (1.4% of the total) were set to missing for these two variables, with four fish having either missing initial or final weights. Furthermore, missing was imposed for fish with an extremely low growth rate (*N* = 21) (relative weight gain less than 6.4%, corresponding to a growth rate of less than 1.3 g) or an extremely high growth rate (*N* = 7) (relative weight gain higher than 49%, not accompanied by a corresponding change in the isotope profile), indicating abnormal development and phenotyping error, respectively.

From the tissue samples, the following Atom% variables were available at the individual level: Atom% for ^13^C in muscle (AMC_*i*_), ^15^N in muscle (AMN_*i*_), ^13^C in liver (ALC_*i*_), ^15^N in liver (ALN_*i*_) and ^13^C in adipose tissue (AAC_*i*_). Lack of tissue sample resulted in nine fish with missing records for Atom% variables; AAC (5), AMC (1), AMN (1), ALC (1) and ALN (1).

From feed recording at the tank level ($t$ = 1…46), tank feed intake ($FI_{t}$, g dry matter) was obtained, as well as the feed conversion ratio ($FCR_{t}$), which calculated as follows:

$$FCR_{t} = \frac{{FI_{t} }}{{WG_{t} }},$$

where $WG_{t}$ is the total $WG$ in tank $t$. As mentioned above, 32 fish had missing phenotypes for weight gain and thus were not included in the FCR calculation. Some of these fish had a low or even negative growth indicating that their contribution to the total tank feed intake was likely rather small. In any case, the fraction of fish that lacked growth records was low (< 1.4%), which implies that the potential bias in FCR is limited.

From the individual levels of Atom% ^13^C ($AMC_{i}$) and Atom% ^15^N ($AMN_{i}$) in muscle, individual isotope-based indicator ratio traits for feed conversion ratio ($IFCR$) and feed efficiency ratio ($IFER$); $IFCR\_AMC_{i}$, $IFCR\_AMN_{i}$, $IFER\_AMC_{i}$, and $IFER\_AMN_{i}$, were defined as follows (taking ^15^N as an example):

$$\begin{aligned} IFCR\_AMN_{i} & = \frac{{FW_{i} *APE_{Ni} }}{{FW_{i} - IW_{i} }}, \\\\ IFER\_AMN_{i} & = \frac{{FW_{i} - IW_{i} }}{{FW_{i} * APE_{Ni} }}, \\ \end{aligned}$$

where $APE_{Ni} = (AMN_{i} - IA \% )$ with $IA \%$ equal to 0.370% for ^15^N and 1.087% for ^13^C. After diet switching, the APE of a stable isotope in muscle tissue is expected to be proportional to the fraction of newly synthesized nutrients in the muscle, and the product of APE and final weight is expected to be proportional to the mass of new nutrients in body tissue. Because the $IFCR$ ratio is expected to be proportional to the amount of newly deposited body nutrients per g increase in body weight, fish that exchange a larger fraction of the body mass per unit of growth will be less feed-efficient. Exchange of body tissue is traceable with stable-isotope profiling and is related to the feed intake of the individual, while the denominator of the ratio is the weight gain, and the ratio between these two variables is equal to $IFCR$ or, alternatively, the inverse is equal to $IFER$.

### Statistical analysis of FCR

At the tank level, first we examined to what degree tank averages for $\overline{WG}$ and $\overline{RG}$, in addition to the tank average isotope content, could explain variation in $FCR$ between tanks by using the following multiple regression model:

$$FCR_{td} = \mu + \beta d + bX_{t} + e_{t} ,$$

where $FCR_{td}$ is the observed FCR in tank $t$ on sampling-day $d$, the latter taking values 1 to 5 and was included as a covariate, since this gave better predictive ability, $\beta$ is the corresponding regression coefficient, $\it {\text{X}}_{t}$ is the covariate value for tank $t$ based on one of the following covariates at a time: $FI$, $\overline{WG}$, $\overline{RG}$, $\overline{AMC}$, $\overline{AMN}$, $\overline{ALC}$, $\overline{ALN}$ and $\overline{AAC}$, $b$ is the corresponding regression coefficient, and $e_{t}$ is the tank residual. The final model was chosen by including the covariates: $FI$, $\overline{RG}$, $\overline{AMC}$, $\overline{AMN}$, $\overline{ALC}$, $\overline{ALN}$ and $\overline{AAC}$ ($k = 7$) simultaneously using the following model:

$$FCR_{td} = \mu + \beta d + \mathop \sum \limits_{j = 1}^{k} b_{j} X_{jt} + e_{t} .$$

Backward elimination with leave-one-out cross-validation was used to identify the model with the lowest predicted residual error sum of squares (PRESS). The analyses were conducted using PROC REG in SAS^®^.

For all regression models, the bias of the model was calculated as the average difference between the observed phenotypes and predicted values obtained by PROC GLM in SAS^®^. Moreover, the coefficient of determination of prediction was computed as:

$$\hat{R}^{2} = 1 - \frac{PRESS}{{SS_{tot} }},$$

where $PRESS = \sum \left( {y_{t} - \hat{y}_{t} } \right)^{2}$ and $\hat{y}_{t}$ is the predicted $FCR$ phenotype for tank $t$, using data from all other tanks in the analysis and $SS_{tot}$ is the total sums of squares. The $\hat{R}^{2}$ is an estimate of the fraction of variance in $FCR$ explained by the model in the prediction of missing observations.

### Genetic analysis

Genetic analysis of traits was performed using the ASReml4 software package [29]. Bivariate analyses were conducted between $FCR$ and $FI$ and of $FCR$ and $FI$ with each of the following traits: $\overline{RG}$, $\overline{WG}$, $\overline{AMC}$, $\overline{AMN}$, $\overline{ALC}$, $\overline{ALN}$, $\overline{AAC}$, $\overline{IFCR\_AMC}$, $\overline{IFCR\_AMN}$, $\overline{IFER\_AMC}$ and $\overline{IFER\_AMN}$. For each bivariate analysis, the model was:

1 $$\left[ {\begin{array}{*{20}c} {{\mathbf{y}_\mathbf{1}} } \\ {\mathbf{y}_\mathbf{2} } \\ \end{array} } \right] = \left[ {\begin{array}{*{20}c} {\mathbf{X}_\mathbf{1} } & \mathbf{0} \\ \mathbf{0} & {\mathbf{X}_\mathbf{2} } \\ \end{array} } \right]\left[ {\begin{array}{*{20}c} {\mathbf{b}_\mathbf{1} } \\ {\mathbf{b}_\mathbf{2} } \\ \end{array} } \right] + \left[ {\begin{array}{*{20}c} {\mathbf{Z}_{\mathbf{a1}} } & \mathbf{0} \\ \mathbf{0} & {{\mathbf{Z}}_{\mathbf{a2}} } \\ \end{array} } \right]\left[ {\begin{array}{*{20}c} {{\mathbf{a}_\mathbf{1}} } \\ {{\mathbf{a}_\mathbf{2}} } \\ \end{array} } \right] + \left[ {\begin{array}{*{20}c} {{\mathbf{e}_\mathbf{1}} } \\ {{\mathbf{e}_\mathbf{2}} } \\ \end{array} } \right],$$

where ${\mathbf{y}}_{1}$ is a vector of tank level phenotypes for $FCR$ or $FI$, ${\mathbf{y}}_{2}$ is a vector of (tank) phenotypes for one of the other traits; $\overline{RG}$, $\overline{WG}$, $\overline{AMC}$, $\overline{AMN}$, $\overline{ALC}$, $\overline{ALN}$, $\overline{AAC}$, $\overline{IFCR\_AMC}$, $\overline{IFCR\_AMN}$, $\overline{IFER\_AMC}$ and $\overline{IFER\_AMN}$, ${\mathbf{b}}_\mathbf{1}$ and ${\mathbf{b}}_\mathbf{2}$ are vectors of fixed effects, including trait-specific intercepts and effects of sampling day, $$\left[ {\begin{array}{*{20}c} {{\mathbf{a}}_\mathbf{1} } \\ {{\mathbf{a}}_\mathbf{2} } \\ \end{array} } \right] \sim N\left( {\mathbf{0},{\mathbf{T}}_\mathbf{0} \otimes {\mathbf{G}}_{{\mathbf{T}}} } \right)$$ is a vector of random additive genetic tank effects for the two traits, $$\left[ {\begin{array}{*{20}c} {{\mathbf{e}}_\mathbf{1} } \\ {{\mathbf{e}}_\mathbf{2} } \\ \end{array} } \right] \sim N\left( {\mathbf{0},{\mathbf{R}} \otimes {\mathbf{I}}} \right)$$ is a vector of random tank residuals for the two traits. The ${\mathbf{X}}$ and ${\mathbf{Z}}$ matrices are appropriate incidence matrices, ${\mathbf{T}}_{0}$ is an additive genetic (co)variance matrix between traits at the tank level, ${\mathbf{G}}_{{\mathbf{T}}}$ is an (46 × 46) additive genetic relationship matrix that describes the average genomic relationships between fish in different tanks and ${\mathbf{R}}$ is the tank residual (co)variance matrix, which was diagonal. Matrix ${\mathbf{G}}_{{\mathbf{T}}}$ was calculated based on a subset of 51,543 SNPs of high genotype quality, covering all chromosomes and is defined as:

$${\mathbf{G}}_{{\mathbf{T}}} = {\mathbf{TT}}\varvec{',}$$

where element $tj$ in ${\mathbf{T}}$ (tank $t$, locus $j$) is: $T_{tj} = \frac{1}{{n_{t} }}\sum\nolimits_{i = 1}^{{n_{t} }} {\left( {M_{ij} - 2P_{j} } \right)}$, $M_{ij}$ is the genotype of individual $i$ within tank $t$ at locus *j*, $P_{j}$ is the allele frequency at locus $j$, and $n_{t}$ is the number of individuals in tank $t$. Finally, the elements of ${\mathbf{G}}_{{\mathbf{T}}}$ were scaled such that the average of the diagonal elements in ${\mathbf{G}}_{{\mathbf{T}}}$ equalled 1.0. Narrow-sense heritability cannot be estimated for traits that are modelled at the tank level, i.e. $FCR$ and $FI$. Instead $h_{t}^{2}$, which quantifies the fraction of the between-tank variance explained by genetics, was estimated as $h_{t}^{2} = \frac{{\sigma_{{a_{t} }}^{2} }}{{\sigma_{{a_{t} }}^{2} + \sigma_{{e_{t} }}^{2} }}$, where $\sigma_{{a_{t} }}^{2}$ ${\text{and }}\sigma_{{e_{t} }}^{2}$ are the estimates at the tank level of additive genetic and residual variance, respectively, of the trait.

The individual phenotypes for $RG$, $WG$, $AMC$, $AMN$, $ALC$, $ALN$, $AAC$, $IFCR\_AMC$, $IFCR\_AMN$, $IFER\_AMC$, and $IFER\_AMN$ were also analysed using bivariate models. For each bivariate analysis, the model was:

2 $$\left[ {\begin{array}{*{20}c} {{\mathbf{y}}_\mathbf{1} } \\ {{\mathbf{y}}_\mathbf{2} } \\ \end{array} } \right] = \left[ {\begin{array}{*{20}c} {{\mathbf{X}}_\mathbf{1} } & \mathbf{0} \\ \mathbf{0} & {{\mathbf{X}}_\mathbf{2} } \\ \end{array} } \right]\left[ {\begin{array}{*{20}c} {{\mathbf{b}}_\mathbf{1} } \\ {{\mathbf{b}}_\mathbf{2} } \\ \end{array} } \right] + \left[ {\begin{array}{*{20}c} {{\mathbf{Z}}_{{{\mathbf{a1}}}} } & \mathbf{0} \\ \mathbf{0} & {{\mathbf{Z}}_{{{\mathbf{a2}}}} } \\ \end{array} } \right]\left[ {\begin{array}{*{20}c} {{\mathbf{a}}_\mathbf{1} } \\ {{\mathbf{a}}_\mathbf{2} } \\ \end{array} } \right] + \left[ {\begin{array}{*{20}c} {{\mathbf{Z}}_{{{\mathbf{t1}}}} } & 0 \\ 0 & {{\mathbf{Z}}_{{{\mathbf{t2}}}} } \\ \end{array} } \right]\left[ {\begin{array}{*{20}c} {{\mathbf{t}}_\mathbf{1} } \\ {{\mathbf{t}}_\mathbf{2} } \\ \end{array} } \right] + \left[ {\begin{array}{*{20}c} {{\mathbf{e}}_\mathbf{1} } \\ {{\mathbf{e}}_\mathbf{2} } \\ \end{array} } \right],$$

where $$\left[ {\begin{array}{*{20}c} {{\mathbf{y}}_{1} } \\ {{\mathbf{y}}_{2} } \\ \end{array} } \right]$$ is a vector of individual phenotypes for the two traits analysed, ${\mathbf{b}}_\mathbf{1}$ and ${\mathbf{b}}_\mathbf{2}$ are vectors of fixed effects for the two traits as described above, $$\left[ {\begin{array}{*{20}c} {{\mathbf{a}}_\mathbf{1} } \\ {{\mathbf{a}}_\mathbf{2} } \\ \end{array} } \right] \sim N\left( {\mathbf{0},{\mathbf{G}}_\mathbf{0} \otimes {\mathbf{G}}} \right)$$ is a vector of random additive genetic effects for the two traits, $$\left[ {\begin{array}{*{20}c} {{\mathbf{t}}_\mathbf{1} } \\ {{\mathbf{t}}_\mathbf{2} } \\ \end{array} } \right] \sim N\left( {\mathbf{0},{\mathbf{T}} \otimes {\mathbf{I}}} \right)$$ is a vector of random tank effects for the two traits, and $$\left[ {\begin{array}{*{20}c} {{\mathbf{e}}_\mathbf{1} } \\ {{\mathbf{e}}_\mathbf{2} } \\ \end{array} } \right] \sim N\left( {\mathbf{0},{\mathbf{R}} \otimes {\mathbf{I}}} \right)$$ is a vector of random residuals. The ${\mathbf{X}}$ and ${\mathbf{Z}}$ matrices are corresponding incidence matrices, ${\mathbf{G}}_\mathbf{0}$ is an additive genetic (co)variance matrix, ${\mathbf{G}}$ is the genomic relationship matrix, ${\mathbf{T}}$ is the tank (co)variance matrix, and ${\mathbf{R}}$ is the residual (co)variance matrix. The genomic relationship matrix was generated according to VanRaden’s first method [30] and was used to account for stratification of the individuals by selection of families based on fast and slow growth rates (in seawater). Matrix ${\mathbf{G}}$ was calculated based on the same subset of SNPs as defined for ${\mathbf{G}}_{{\mathbf{T}}}$ above.

Heritabilities of individual traits were estimated as: $h^{2} = \frac{{\sigma_{a}^{2} }}{{\sigma_{a}^{2} + \sigma_{t}^{2} + \sigma_{e}^{2} }}$, where $\sigma_{a}^{2}$, $\sigma_{t}^{2} , {\text{and }}\sigma_{e}^{2}$ are the estimates of the individual additive genetic, tank environmental, and individual residual variance, respectively, of the trait. The fraction of variance explained by tank was estimated as: $c^{2} = \frac{{\sigma_{t}^{2} }}{{\sigma_{a}^{2} + \sigma_{t}^{2} + \sigma_{e}^{2} }}$. Significance of the genetic effect was tested using a likelihood-ratio ($LR$) test-statistic, comparing a single-trait model with genetic effects (${\text{H}}_{1}$) to a model without genetic effects (${\text{H}}_{0}$) with the ${\mathbf{G}}$ matrices (${\mathbf{G}}_{{\mathbf{T}}}$ and ${\mathbf{G}}$, respectively) in ${\text{H}}_{1}$:

$$LR = 2\left( {\left( {\log L |\hat{\theta }_{{H_{1} }} } \right) - \left( {\log L |\hat{\theta }_{{H_{0} }} } \right)} \right).$$

The genetic effect was considered significant if $LR < \chi_{{\left( {\alpha = 0.05; \,df = 1} \right)}}^{2}$.

## Results

**Fig. 1:**
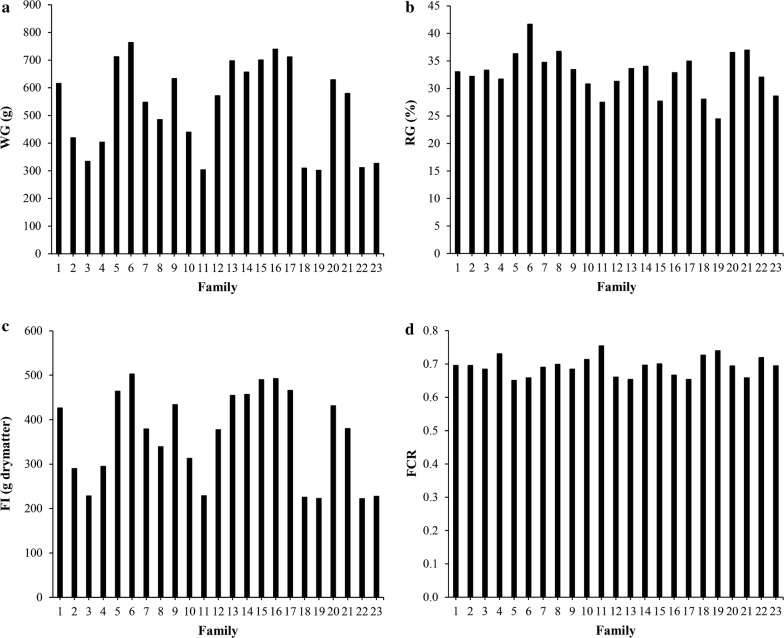
Averages per family for **a** weight gain (WG), **b** relative weight gain (RG), **c** feed intake (FI), and **d** feed conversion ratio (FCR = FI/WG)

**Fig. 2:**
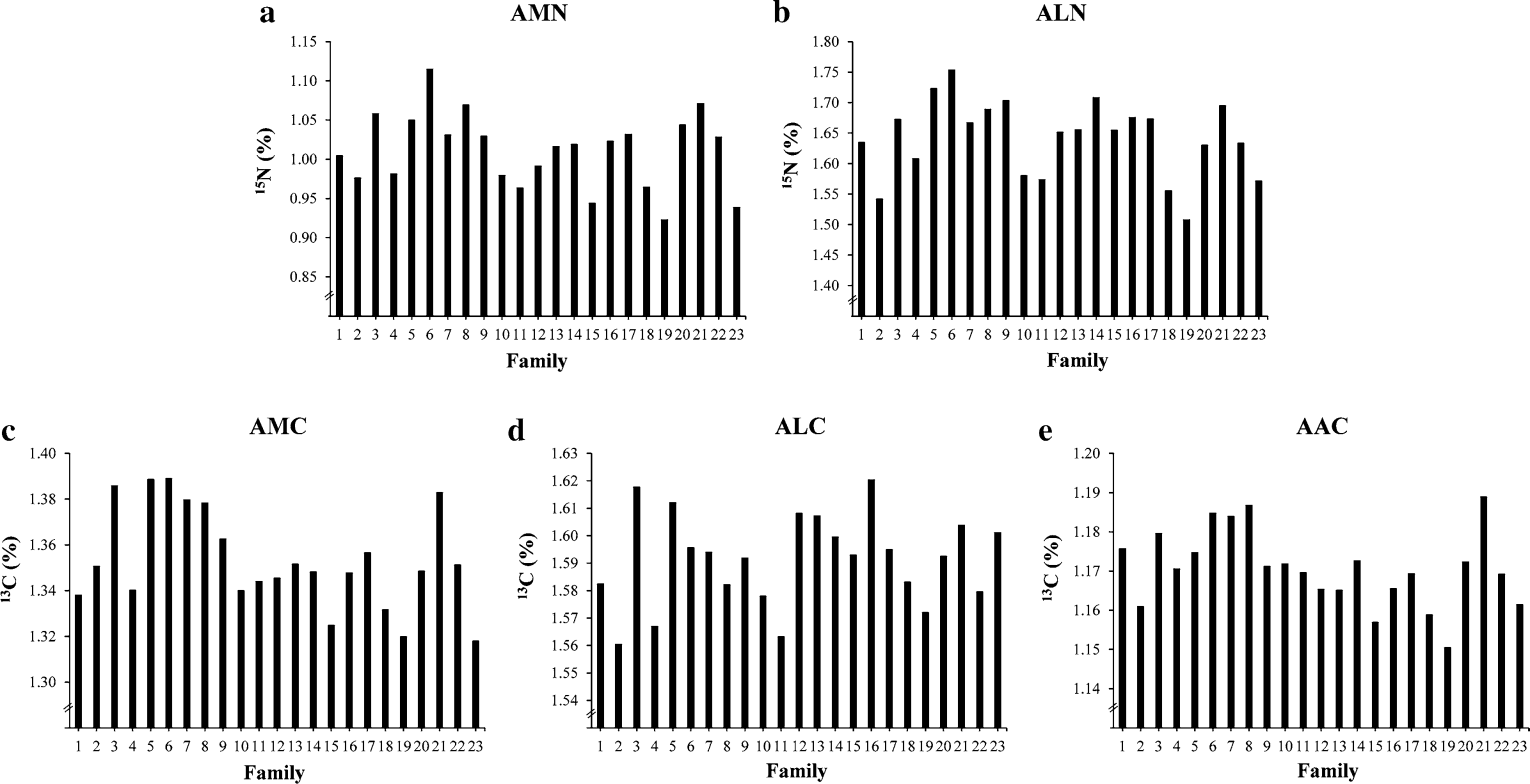
Averages per family for **a** Atom% ^15^N in muscle (AMN), **b** Atom% ^15^N in liver (ALN), **c** Atom% ^13^C in muscle (AMC), **d** Atom% ^13^C in liver (ALC), and **e** Atom% ^13^C in adipose tissue (AAC)

The diet was formulated for increased ^15^N and ^13^C isotope levels, by using 2% and 1% of ^15^N- and ^13^C-labelled spirulina, respectively, which resulted in an Atom% of 2.7 and 2.0 of ^15^N and ^13^C, respectively, in the diet (Table 1). All fish were healthy throughout the experiment and tanks were fed the diet at 10% in excess of uneaten feed. Table 2 shows the descriptive statistics of the data. The mean Atom% of ^15^N and ^13^C in muscle, liver, and adipose tissue ranged from 1.01 to 1.64% and from 1.17 to 1.59%, respectively. These results confirm that none of the tissues was in equilibrium with the diet that contained 2.7 and 2.0% ^15^N and ^13^C, respectively. Thus, variation in the Atom % of ^15^N and ^13^C could be determined between individuals. For the individually recorded traits, large differences in WG and RG were observed between families (Fig. 1a, b). However, for the tank-recorded traits, i.e. FI and FCR, larger differences were observed between families for FI than for FCR (Fig. 1c, d), which was reflected in the larger coefficient of variation for FI than for FCR (Table 2). This is logical because the coefficient of variation for FCR contains the standard deviation for WG, which was calculated from individual observations. Figure 2a–e show the Atom% of ^15^N and ^13^C in muscle, liver, and adipose tissue for all families, showing considerable differences between families.

**Table 2:** Descriptive statistics of recorded trait phenotypes

Trait name	Abbreviation	Mean	Min	Max	SD	CV
*Individual traits* (*N = 2281*)						
Initial weight (g)	IW	21.8	1.7	52.4	8.0	36.8
Final weight (g)	FW	32.6	4.9	70.3	11.3	34.8
Weight gain: ($FW{-}IW$) (g)	WG	10.8	0.3	30.6	4.5	41.9
Relative weight gain: ($\left( {\left( {FW{-}IW} \right)/FW} \right) \times 100$) (%)	RG	32.8	1.8	64.3	8.1	24.6
Atom% ^13^C in muscle (%)	AMC	1.35	1.14	1.62	0.05	3.8
Atom% ^15^N in muscle (%)	AMN	1.01	0.54	1.76	0.12	11.8
Atom% ^13^C in liver (%)	ALC	1.59	1.27	1.77	0.04	2.4
Atom% ^15^N in liver (%)	ALN	1.64	0.77	2.00	0.13	7.9
Atom% ^13^C in adipose tissue (%)	AAC	1.17	1.09	1.55	0.02	2.1
*Tank traits* (*N = 46*)						
Feed intake (g dry matter)^a^	FI	363	163	556	110	30.0
Feed conversion ratio: ($FI/FW - IW$)	FCR	0.69	0.64	0.78	0.03	4.8

^a^Calculated according to Helland et al. [23]

Table 3 shows that $\overline{RG}$ explained the largest fraction of variance in FCR as a single variable (in addition to day) ($R^{2}$ = 62% and $\hat{R}^{2}$ = 55%), followed by $\overline{ALC}$ ($R^{2}$ = 57% and $\hat{R}^{2}$ = 52%) and $\overline{WG}$ ($R^{2}$ = 53% and $\hat{R}^{2}$ = 46%). When simultaneously regressing all the explanatory variables on FCR and using backward elimination, the preferred model with the lowest PRESS value had an $R^{2}$ of 79% (Table 3). This implies that the variables included in the model explained a major part of the variation between tanks with respect to FCR. The variables retained were Day, $\overline{RG}$, $\overline{AMN}$, $\overline{ALC}$ and $\overline{AAC}$. Using leave-one-out cross-validation, the coefficient of determination of the predicted tank averages was $\hat{R}^{2}$ = 73%, i.e., even when predicting missing observations, the model explained most of the tank variation in FCR, while the bias was negligible. Moreover, when including interactions between indicator variables in the backward elimination process (data not shown), PRESS was reduced to 0.0118 in the preferred model, which had an $R^{2}$ of 88%, while $\hat{R}^{2}$ was 77% under prediction.

**Table 3:** Results of regression analysis of tank level feed conversion rate on sampling day and each indicator trait, one by one, or when regressing on all^a^ experimental variables, following backward elimination

Indicator trait(s)	R^2^	Adjusted-R^2^	$\hat{R}^{2}$ ^b^	Bias	PRESS^c^
FI + Day	0.42	0.39	0.32	4.3 × 10^−10^	0.035
$\overline{WG}$ + Day	0.53	0.51	0.46	6.5 × 10^−10^	0.028
$\overline{RG}$ + Day	0.62	0.60	0.55	2.2 × 10^−10^	0.023
$\overline{AMC}$ + Day	0.31	0.28	0.21	− 4.4 × 10^−10^	0.041
$\overline{AMN}$ + Day	0.42	0.40	0.34	− 4.4 × 10^−10^	0.034
$\overline{ALC}$ + Day	0.57	0.55	0.52	− 4.4 × 10^−10^	0.025
$\overline{ALN}$ + Day	0.49	0.46	0.40	− 4.4 × 10^−10^	0.031
$\overline{AAC}$ + Day	0.16	0.12	0.03	− 4.4 × 10^−10^	0.050
$\overline{RG}$ + $\overline{AMN}$ + $\overline{ALC}$ + $\overline{AAC}$ + Day^d^	0.79	0.77	0.73	0.00	0.014

^a^Except weight gain

^b^$\hat{R}^{2}$ = The coefficient of determination (R^2^)

^c^PRESS = predicted residual error sums of squares

^d^All variables left in the model are significant at the 0.10 level

The results obtained for traits recorded at the tank level and analysed with model (1) showed that genetic background (family) explained 52 and 92% of the between-tank variation for FCR (*p* = 0.0002) and FI (*p* = 9.3 × 10^−16^), respectively (Table 4), i.e., the corresponding correlations between the average family phenotypes in different tanks were 0.72 and 0.96 for FCR and FI, respectively. For the individually recorded traits, significant (*p* < 0.05) heritabilities were estimated for all traits. The estimated heritability for WG was high (0.45), whereas heritabilities were moderate for RG, AMC, AMN, ALC, ALN and AAC (0.28, 0.18, 0.28, 0.15, 0.26 and 0.18, respectively), and relatively low for IFCR_AMC, IFCR_AMN, IFER_AMC and IFER_AMN (0.09, 0.06, 0.11 and 0.08, respectively). Non-genetic tank effects were generally low and explained 2 to 13% of the total phenotypic variance for individual traits.

**Table 4:** Estimates with standard errors of genetic and residual variance components ($\sigma_{a}^{2}$ and $\sigma_{e}^{2}$, respectively), fraction of phenotypic variance explained by environmental tank effect ($c^{2}$), heritability ($h^{2}$), fraction of between-tank variance explained by genetics ($h_{t}^{2}$), as well as the *χ*^2^ statistics for the additive genetic family effect, with the corresponding level of significance (*p*)

	$\sigma_{a}^{2}$ ^a^	$\sigma_{e}^{2}$ ^a^	$c^{2}$	$h^{2}$	$h_{t}^{2}$	$\it \chi^{2}$	*p*
FCR	5.48 ± 2.69	4.63 ± 1.49	–	–	0.52 ± 0.17	14.0	0.0002
FI	50.99 ± 16.50	4.68 ± 1.87	–	–	0.92 ± 0.04	64.6	9.3 × 10^−16^
WG	5.82 ± 0.67	6.44 ± 0.26	0.06 ± 0.02	0.45 ± 0.04	–	222.6	2.4 × 10^−50^
RG	14.42 ± 2.36	36.35 ± 1.34	0.03 ± 0.01	0.28 ± 0.04	–	106.7	5.1 × 10^−25^
AMC	4.62 ± 0.97	19.43 ± 0.68	0.05 ± 0.02	0.18 ± 0.03	–	47.5	5.4 × 10^−12^
AMN	39.23 ± 6.38	97.32 ± 3.58	0.02 ± 0.01	0.28 ± 0.04	–	101.2	8.1 × 10^−24^
ALC	1.90 ± 0.44	9.14 ± 0.32	0.13 ± 0.04	0.15 ± 0.03	–	43.1	5.2 × 10^−11^
ALN	40.99 ± 7.09	110.20 ± 4.04	0.05 ± 0.02	0.26 ± 0.04	–	77.0	1.7 × 10^−18^
AAC	0.99 ± 0.21	4.21 ± 0.15	0.05 ± 0.02	0.18 ± 0.03	–	46.0	1.2 × 10^−11^
IFCR_AMC	28.88 ± 8.83	275.38 ± 9.18	0.03 ± 0.01	0.09 ± 0.03	–	24.7	6.6 × 10^−07^
IFCR_AMN	86.92 ± 35.33	1270.90 ± 41.67	0.04 ± 0.02	0.06 ± 0.02	–	13.0	0.0003
IFER_AMC	45.75 ± 12.92	364.42 ± 12.28	0.05 ± 0.02	0.11 ± 0.03	–	29.4	5.9 × 10^−08^
IFER_AMN	3.59 ± 1.22	39.72 ± 1.32	0.04 ± 0.02	0.08 ± 0.02	–	21.3	4.0 × 10^−06^

^a^Variance components and standard error estimates have been multiplied with 10^4^, except WG and RG

Genetic correlations between FCR/FI and all the other traits were estimated with model (1) and those between the remaining traits were estimated with model (2) (Table 5). Generally, estimates of the genetic correlation between FCR, measured at the tank level, with each other trait were negative, while those for the IFCR were positive, as expected. This means that FI, growth (WG and RG), and the indicator traits (i.e. the fraction of newly deposited tissue) all had favourable genetically correlations with FCR. For the indicator traits measured directly (excluding the indicator ratio traits), the closest genetic correlation with FCR was estimated for ALC (− 0.90 ± 0.11), followed by RG (− 0.82 ± 0.10), WG (− 0.74 ± 0.17), AMN (− 0.73 ± 0.14), AMC (− 0.69 ± 0.17), ALN (− 0.63 ± 0.19), FI (− 0.61 ± 0.21), and AAC (− 0.43 ± 0.28). In addition, a perfect genetic correlation was estimated between the indicator ratio traits IFCR_AMC, IFCR_AMN and IFER_AMN and FCR (1.0, 1.0 and − 1.0), except for IFER_AMC, which had a lower genetic correlation estimate with FCR (− 0.63 ± 0.30), albeit not significantly different from 1. Internally, IFCR and IFER variables had high estimated genetic correlations (− 0.71 to − 0.99). In general, estimated genetic correlations of the isotope content of the various tissues with growth (in particular RG) and FI were positive. Among the indicator traits, ALC had the lowest genetic correlation with the other isotope indicator traits (0.04–0.38) and with RG (0.12). AMN and ALN were closely genetically correlated to each other (0.89), which indicates that nitrogen metabolism in liver and in muscle are largely the same genetic trait. Estimates of the genetic correlation of AMN and ALN with RG were high (0.98 and 0.89, respectively). Likewise, AMC and AAC were closely genetically correlated with each other (0.73), with ALN and AMN (0.69 to 0.96) and with RG (0.78 to 0.92). FI was also closely genetically correlated with WG (0.98). For individual traits, phenotypic and genetic correlations were generally similar. Among the traits evaluated, ALC, IFCR (for both nitrogen and carbon) and IFER (for nitrogen) stood out as individual indicator traits for FCR. Estimates of the genetic correlation of ALC with the indicator ratio traits IFCR and IFER were low for both nitrogen and carbon (− 0.27 to 0.11).

**Table 5:** Estimates of genetic (above diagonal) and phenotypic (below diagonal) correlations between traits, with standard errors

Traits	FCR^a^	FI	WG	RG	AMC	AMN	ALC	ALN	AAC	IFCR_AMC	IFCR_AMN	IFER_AMC	IFER_AMN
FCR		− 0.61 ± 0.21	− 0.74 ± 0.17	− 0.82 ± 0.10	− 0.69 ± 0.17	− 0.73 ± 0.14	− 0.90 ± 0.11	− 0.63 ± 0.19	− 0.43 ± 0.28	1.0^b^	1.0^b^	− 0.63 ± 0.30	− 1.0^b^
FI	− 0.52 ± 0.13		0.98 ± 0.01	0.44 ± 0.20	0.13 ± 0.24	0.16 ± 0.23	0.31 ± 0.25	0.40 ± 0.21	0.13 ± 0.26	− 0.79 ± 0.17	− 0.91 ± 0.21	0.76 ± 0.22	0.84 ± 0.16
WG	− 0.65 ± 0.10	0.97 ± 0.01		0.46 ± 0.07	0.19 ± 0.11	0.28 ± 0.09	0.16 ± 0.12	0.56 ± 0.07	0.44 ± 0.04	− 0.76 ± 0.1	− 0.83 ± 0.15	0.75 ± 0.09	0.74 ± 0.12
RG	− 0.79 ± 0.07	0.45 ± 0.16	0.54 ± 0.02		0.92 ± 0.04	0.98 ± 0.01	0.12 ± 0.14	0.89 ± 0.03	0.78 ± 0.07	− 0.83 ± 0.08	− 0.80 ± 0.11	0.76 ± 0.09	0.74 ± 0.1
AMC	− 0.61 ± 0.12	0.17 ± 0.19	0.29 ± 0.03	0.71 ± 0.01		0.96 ± 0.02	0.38 ± 0.14	0.88 ± 0.04	0.73 ± 0.09	− 0.61 ± 0.17	− 0.65 ± 0.16	0.47 ± 0.18	0.57 ± 0.16
AMN	− 0.70 ± 0.1	0.22 ± 0.19	0.35 ± 0.03	0.82 ± 0.009	0.86 ± 0.008		0.20 ± 0.14	0.89 ± 0.03	0.71 ± 0.08	− 0.72 ± 0.12	− 0.72 ± 0.15	0.63 ± 0.13	0.63 ± 0.15
ALC	− 0.73 ± 0.08	0.25 ± 0.18	0.09 ± 0.03	0.19 ± 0.03	0.20 ± 0.03	0.15 ± 0.03		0.34 ± 0.12	0.04 ± 0.16	0.10 ± 0.20	− 0.27 ± 0.22	− 0.26 ± 0.18	0.11 ± 0.20
ALN	− 0.63 ± 0.12	0.39 ± 0.17	0.42 ± 0.03	0.74 ± 0.01	0.63 ± 0.02	0.70 ± 0.01	0.49 ± 0.02		0.69 ± 0.08	− 0.70 ± 0.11	− 0.77 ± 0.12	0.58 ± 0.13	0.66 ± 0.13
AAC	− 0.40 ± 0.16	0.17 ± 0.19	0.30 ± 0.03	0.60 ± 0.02	0.52 ± 0.02	0.56 ± 0.02	0.24 ± 0.03	0.54 ± 0.02		− 0.58 ± 0.14	− 0.63 ± 0.15	0.53 ± 0.14	0.62 ± 0.14
IFCR_AMC	0.58 ± 0.11	− 0.56 ± 0.12	− 0.41 ± 0.02	− 0.58 ± 0.02	0.06 ± 0.03	− 0.19 ± 0.03	− 0.12 ± 0.03	− 0.37 ± 0.02	− 0.25 ± 0.02		0.90 ± 0.006	− 0.96 ± 0.03	− 0.89 ± 0.07
IFCR_AMN	0.72 ± 0.09	− 0.54 ± 0.11	− 0.41 ± 0.02	− 0.57 ± 0.02	0.10 ± 0.03	− 0.10 ± 0.03	− 0.21 ± 0.03	− 0.36 ± 0.02	− 0.26 ± 0.02	0.87 ± 0.007		− 0.71 ± 0.13	− 0.99 ± 0.03
IFER_AMC	− 0.46 ± 0.14	0.50 ± 0.13	0.42 ± 0.02	0.58 ± 0.02	− 0.14 ± 0.03	0.21 ± 0.03	0.07 ± 0.03	0.33 ± 0.02	0.24 ± 0.03	− 0.87 ± 0.006	− 0.66 ± 0.01		0.82 ± 0.09
IFER_AMN	− 0.74 ± 0.08	0.57 ± 0.11	0.45 ± 0.02	0.62 ± 0.02	− 0.10 ± 0.03	0.09 ± 0.03	0.16 ± 0.03	0.34 ± 0.02	0.28 ± 0.02	− 0.77 ± 0.01	− 0.88 ± 0.005	0.74 ± 0.01	

^a^Genetic analysis with FCR and FI was conducted on a tank basis, due to lack of individual recording for FCR and FI (*N* = 46)

^b^Restricted on boundary of parameter space

## Discussion

In aquaculture, feed constitutes about half of the total production costs in the grow-out phase at sea [31]. Genetic improvement of feed efficiency will reduce production costs and, at the same time, have a favourable environmental impact by maximizing resource utilization and reducing nutrient load (e.g., nitrogen) to the environment. Protein metabolism is a major determinant of the conversion of feed into growth. Consequently, minimizing the energetic cost of protein metabolism is a strategic goal for enhancing fish growth and feed efficiency. Because Atom% of nitrogen and carbon, and functions thereof, can be individually recorded, these traits could be used as indicator traits for individual feed conversion ratio in growing fish.

The observed between-family difference in nitrogen and carbon metabolism (Fig. 2a–e) have the potential to affect feed efficiency. To evaluate whether ^15^N and ^13^C stable isotopes can be used to capture variation in feed efficiency in Atlantic salmon, the observed tank level FCR was best predicted using a multiple regression model that included $\overline{RG}$, $\overline{AMN}$, $\overline{ALC}$ and $\overline{AAC}$ as covariates, in addition to sampling day. This prediction model explained 73% of the variation in masked FCR records (Table 3; $\hat{R}^{2}$). The single most important isotope variable for prediction was $\overline{ALC}$, which together with sampling day explained 57% ($R^{2}$) of the variation in FCR (Table 3). In comparison, for similar models using $\overline{WG}$ or $\overline{RG}$, the proportions of variance in FCR explained were 53 and 62%, respectively (Table 3). Hence, by including information on isotope profiles, prediction of FCR data was substantially improved, compared to what was obtained by growth data alone (Table 3). This indicates that stable isotopes can be used to improve the prediction of individual FCR, which is of considerable value to fish breeding. The regression analyses revealed that, after adjusting for growth, improved feed efficiency was associated with reduced metabolism of nitrogen in muscle (AMN, β = 0.31 ± 0.17, results not shown) and reduced carbon metabolism in adipose tissue (AAC, β = 0.90 ± 0.41), but with elevated carbon metabolism in liver (ALC, β = − 0.75 ± 0.18). In fish, the main source of nitrogen in both liver and muscle is protein [7]. Similarly, the main sources of carbon in muscle is protein, but in liver the main sources are protein, fat and glycogen [32]. In adipose tissue, the main source of carbon is lipids, but they can originate from lipid biosynthesis from protein through oxidative degradation and deamination of amino acids, or from carbohydrates through acetyl-CoA formed in the mitochondria [33].

Fish are highly efficient in converting dietary protein into body protein [34]. This requires regulation of the flux of amino acids into metabolic fates such as oxidation, gluconeogenesis, and lipogenesis. Because salmonids are carnivores, they depend highly on glucose synthesis from non-carbohydrate sources. The surplus of amino acids has a major role in energy metabolism as oxidative substrates in many tissues. Fish with efficient growth seem to use a low-protein metabolism strategy [9, 35, 36]. It has also been reported that a reduced capacity for body lipid deposition is favourably associated with high protein growth efficiency [12]. Furthermore, low proteasome activity, i.e., reduced proteolysis in the liver has been linked to higher growth efficiency [37]. In this experiment, adjusted for growth, efficient fish were characterized by older nitrogen (i.e. protein) profiles of muscle tissues, which confirms earlier findings, since reduced proteolysis of body protein will preserve more of the old protein. These results suggest that efficient fish had newer liver carbon profiles (i.e., glycogen, fat, and protein, combined), which might be linked to the origin of the glycogen in the liver; efficient fish possibly synthesize relatively more of their glycogen through gluconeogenesis or lipogenesis in the liver and thus from nutrients that come directly from digestion of feed (new nutrients) and relatively less from proteolysis of older body protein. Our findings indicate that fast growth combined with reduced degradation rates of existing body tissues, especially in the muscle, is favourable, and that individual differences in these traits can be captured by nitrogen and carbon isotope profiling of the various tissues. The underlying biological mechanisms are likely complex and further studies are needed to elucidate the underlying factors relevant to feed efficiency.

The prediction equation for individual feed efficiency shows that indicator traits add information to the prediction of feed efficiency beyond growth. The limitation of the prediction equation developed is that all variables are averages at the tank level because FCR was only recorded per tank. If this prediction equation was to be used to predict individual FCR, this would imply that the phenotypic and genetic correlations are assumed to be the same at both the individual and group levels, which may not be realistic. In addition, the prediction equation was estimated in freshwater during a phase of high growth and needs to be validated or re-estimated for larger fish in seawater, but this would require recording of feed intake in the sea. A prediction equation estimated during the freshwater phase may not predict the feed efficiency performance in the grow-out phase in the sea very well. During grow-out, other metabolic pathways such as lipid metabolism may explain more of the variation in feed efficiency in Atlantic salmon, since the relative weight gain decreases, which may leave more room for other factors than growth to contribute to feed efficiency, as previously demonstrated for large rainbow trout [12]. Hence, it is considered more effective to use individually measured phenotypes that are highly genetically correlated to feed efficiency to improve the feed efficiency indirectly. Indicator traits that are more highly correlated to feed efficiency in later life-stages could, therefore, be of high value.

Estimates of genetic correlations (Table 5) revealed, as expected, that fast growth (WG and RG) is favourably associated with improved feed efficiency (*r* = − 0.74 and -0.82, respectively). The indicator traits AMN, AMC, and ALN were estimated to be highly genetically correlated with the growth traits and feed efficiency, as expected, since body growth depends on the deposition of new nutrients from enriched feed, which increased isotope levels in tissues. The estimate of the genetic correlation of carbon metabolism in adipose tissue with FCR (− 0.43) was moderate. The link between lipid deposition and FCR should, however, not be disregarded, since it is known to affect feed efficiency later in the life cycle of salmonids because lipid deposition is at its maximum first during the grow-out phase in the sea [12]. ALC had the closest estimated genetic correlation with FCR (− 0.90), but had lower genetic correlation estimates with the other indicator traits, which suggests that ALC might explain additional variation in the feed efficiency complex among the indicator traits considered here. As explained above, protein is likely the main source of nitrogen and carbon in muscle and nitrogen (but not necessarily carbon) in liver. This might explain the high genetic and phenotypic correlations of nitrogen and carbon metabolism in the muscle and nitrogen metabolism in the liver, since they all likely reflect protein metabolism. Compared with muscle, carbon metabolism in the liver (ALC) is affected by fat and glycogen to a larger extent and, thus, is expected to relate less to the other indicator traits.

The IFCR and IFER variables for nitrogen and carbon in muscle are expected to be proportional to the mass of newly deposited nutrients in muscle and, as such, relate directly to the efficiency complex. Buchheister and Latour [38] proposed a ratio between specific growth rate and total metabolism, estimated from isotope profiling, as an indicator trait. A preliminary analysis showed that the trait definition of Buchheister and Latour was close to perfectly genetically correlated with the IFER indicators used in this study (results not shown). In our study, the estimate of the genetic correlation of IFCR with the observed FCR was very high, to the extent that the estimate was fixed at the border of the parameter space ($r_{g} \sim 1.0$) for both nitrogen and carbon metabolism in muscle, with a phenotypic (tank-level) correlation with observed FCR of 0.72 and 0.58, respectively. The IFER_AMN variable, being the inverse of IFCR_AMN, and correspondingly IFER_AMC were estimated with, respectively a highly negative genetic correlation (− 1.0) and a moderately negative, albeit highly uncertain, genetic correlation (− 0.63 ± 0.30) to FCR. These results indicate that the mass of new nutrients in the muscle is closely genetically associated with FCR at the tank level. Since the indicator ratio traits (IFCR/IFER) can be measured on individual fish, they are promising indicator traits for individual phenotyping of feed efficiency. However, the estimates of heritability of the indicator ratio traits were lower (0.06 to 0.11) than the estimates of heritability for the remaining traits. In addition, estimates of the genetic correlation of the indicator ratio traits IFCR_AMC, IFCR_AMN, IFER_AMC, and IFER_AMN with ALC were low. However, estimates of the genetic correlation of ALC and the indicator ratio traits with tank-FCR were high, which indicates that ALC explained individual variation in feed efficiency that was not explained by growth. The indicator ratio traits IFCR and IFER are intuitively appealing and can be easily interpreted biologically, compared to ALC, for which the underlying determinants are largely unknown. The efficiency of metabolization and allocation of nutrients for growth is closely related to the feed efficiency complex; using body tissue as fuel for, e.g., maintenance, is less efficient than using nutrients absorbed and metabolized from feed directly. However, there is some variation between individuals in the extent to which body tissues are used for maintenance [10, 39–41]. A lower exchange of body tissue components would result in more efficient use of protein and thus reduced feed costs [40]. The IFCR and IFER variables allow for direct measurement of nitrogen and carbon fluxes by using stable-isotope profiling to trace the contribution and allocation of nutrients from feed to growth in animal tissue [40–42] and are expected to have a universal relationship with FCR and could be useful independently of life-stage and species.

The standard errors of the estimates of the genetic correlations were rather low in spite of the limited number of families in the study. However, the standard errors of the genetic correlations between our traits and FCR could be made smaller by increasing the size of the family dataset and could thus be used to validate our approach. Our experimental design made it possible to keep all individuals in one common environment until the start of the experiment, which strengthens our results by reducing the environmental variation between families. Our results indicate that the total variation between tanks was, to a large extent, explained by genetics, 52% for FCR and 92% for feed intake.

Phenotyping of stable isotopes at the individual level requires liver and muscle samples, which normally implies that the fish are sacrificed. However, the isotope profile in muscle can be obtained from a muscle biopsy on live animals, which would allow these indicator ratio traits to be recorded even on selection candidates. Alternatively, if test fish have to be sacrificed through sib-testing, information on the full-sibs can be used to predict breeding values on the untested selection candidates. Genomic selection methods that use individual phenotypes and genotypes on training animals for selection among genotyped candidates are expected to be much more effective than traditional pedigree-based selection methods [43–45]. Hence, individual phenotyping is still very important, even for traits that cannot be recorded on the selection candidates. Thus, in full-sib testing an indicator trait is efficient if the estimated breeding value for the indicator ratio trait is estimated with high accuracy (which requires a considerable number of full-sibs), the indicator trait has a high genetic correlation with feed efficiency (as estimated for the IFCR phenotype), and feed efficiency has significant genetic variance (considered considerable, with 3% point standard deviation for FCR). A slaughter test using full-sibs of the breeding candidates is currently part of the breeding program and, thus, implementation of the indicator ratio traits can be carried out in the existing test under field conditions.

## Conclusions

Given that isotope-enriched feed can be produced at an acceptable cost, this study presents indicator ratio traits for individual FCR that might be recorded on a massive scale and used for selection, without requiring individual feed intake recording. This requires that the indicator ratio traits, IFCR and IFER, which have a strong genetic relationship to FCR (as reported here in freshwater) are also shown to have such a genetic relationship in the grow-out phase.
